# Age-Related Effects of Inhalational Anesthetics in *B4galnt1*-Null and Cuprizone-Treated Mice: Clinically Relevant Insights into Demyelinating Diseases

**DOI:** 10.3390/cimb46080494

**Published:** 2024-08-01

**Authors:** Ozana Katarina Tot, Stefan Mrđenović, Vedrana Ivić, Robert Rončević, Jakov Milić, Barbara Viljetić, Marija Heffer

**Affiliations:** 1 Department of Anesthesiology, Resuscitation and Intensive Care, University Hospital Center Osijek, 31000 Osijek, Croatia; oktot@mefos.hr; 2 Department of Anesthesiology, Resuscitation, Intensive Care Medicine and Pain Management, Faculty of Medicine Osijek, Josip Juraj Strossmayer University, 31000 Osijek, Croatia; 3 Department of Hematology, Internal Medicine Clinic, University Hospital Center Osijek, 31000 Osijek, Croatia; mrdenovic@gmail.com; 4 Department of Internal Medicine and History of Medicine, Faculty of Medicine Osijek, Josip Juraj Strossmayer University, 31000 Osijek, Croatia; 5 Department of Medical Biology and Genetics, Faculty of Medicine Osijek, Josip Juraj Strossmayer University, 31000 Osijek, Croatia; vedrana.ivic@mefos.hr (V.I.); milic.jakov@gmail.com (J.M.); mheffer@mefos.hr (M.H.); 6 Department of Diagnostic and Interventional Radiology, University Hospital Center Osijek, 31000 Osijek, Croatia; robert.roncevic27@gmail.com; 7 Department of Medical Chemistry, Biochemistry and Clinical Chemistry, Faculty of Medicine Osijek, Josip Juraj Strossmayer University, 31000 Osijek, Croatia

**Keywords:** inhalational anesthetics, demyelination, *B4galnt1* mice, cuprizone

## Abstract

Anesthetics are essential agents that are frequently used in clinical practice to induce a reversible loss of consciousness and sensation by depressing the central nervous system. The inhalational anesthetics isoflurane and sevoflurane are preferred due to their rapid induction and recovery times and ease of administration. Despite their widespread use, the exact molecular mechanisms by which these anesthetics induce anesthesia are not yet fully understood. In this study, the age-dependent effects of inhalational anesthetics on two demyelination models were investigated: congenital (*B4galnt1*-null) and chemically induced (cuprizone). Various motor and cognitive tests were used to determine sensitivity to isoflurane and sevoflurane anesthesia. *B4galnt1*-null mice, which exhibit severe motor deficits due to defects in ganglioside synthesis, showed significant impairments in motor coordination and balance in all motor tests, which were exacerbated by both anesthetics. Cuprizone-treated mice, which mimic the demyelination in *B4galnt1*-null mice, also showed altered, age-dependent sensitivity to anesthesia. The study showed that older mice exhibited more pronounced deficits, with *B4galnt1*-null mice showing the greatest susceptibility to sevoflurane. These differential responses to anesthetics suggest that age and underlying myelin pathology significantly influence anesthetic effects.

## 1. Introduction

Anesthetics are crucial in both human and veterinary medicine as they facilitate various medical procedures by inducing reversible unconsciousness. Despite their widespread use, the exact mechanisms of action of inhalational anesthetics are still not fully understood. Volatile anesthetics such as isoflurane and sevoflurane are commonly used in the clinic due to their rapid induction and recovery times. Isoflurane, chemically known as 1-chloro-2,2,2-trifluoroethyldifluoromethyl ether, is a clear, colorless liquid with a slightly pungent odor at room temperature. Sevoflurane is chemically known as fluoromethyl 2,2,2-trifluoro-1-(trifluoromethyl) ethyl ether and has similar properties to isoflurane, including in its safety profile.

Although inhalational anesthetics such as isoflurane and sevoflurane can activate GABA_A_ receptors (GABA_A_R) and glycine receptors and inhibit or modulate glutamate, 5-hydroxytryptamine (5-HT), nicotinic acetylcholine receptors and ion channels [1], the precise mechanisms of their action remain incompletely understood. It is hypothesized that 300–3000 cell proteins are potential binding targets for anesthetics [2], indicating the complexity of their potential interactions in the nervous system. Furthermore, it is speculated that anesthetics exert their effects in part by disrupting lipid rafts, membrane regions that are essential for the compartmentalization of membrane proteins and associated molecules. Their disruption can lead to activation of TWIK-related potassium channel-1 (TREK-1), which contributes to the reversible loss of consciousness observed during anesthesia [3]. As mentioned above, lipid rafts are specialized microdomains within the cell membrane that are enriched in cholesterol, sphingolipids and certain proteins. They play a central role in various cellular processes, including signal transduction, protein trafficking and modulation of membrane fluidity [4]. Despite their crucial role in cell physiology, a comprehensive understanding of how inhalational anesthetics interact with these microdomains to induce anesthesia remains elusive.

In particular, hereditary defects in the synthesis and degradation of glycosphingolipids or cholesterol as well as neurodegenerative diseases such as multiple sclerosis, Parkinson’s disease, Alzheimer’s disease and lysosomal storage diseases are characterized by dysregulation of lipid metabolism and lipid rafts [5,6], suggesting that alterations in lipid raft dynamics may influence the sensitivity and efficacy of anesthetics. Induction of general anesthesia in patients with these neurodegenerative diseases may cause different effects and complications than in healthy individuals [7]. Inhalational anesthetics have been associated with the exacerbation of inflammation and neuronal damage in these diseases, and the effects appear to be age-related [8].

In the nervous system, lipid rafts are crucial for maintaining the integrity and functionality of neuronal cells [6], affecting neurotransmitter signaling, and they may have an impact on the mechanism of action of anesthetics. The *B4galnt1* gene encodes the enzyme GM2/GD2 synthase (UDP-N-acetyl-D-galactosamine/GD3 N-acetyl-D-galactosaminyltransferase, EC 2.4.1.92), an enzyme responsible for the biosynthesis of complex gangliosides (GM1, GD1a, GD1b and GT1b), integral components of lipid rafts [9,10,11]. Gangliosides are sialic acid-containing glycosphingolipids and important molecular determinants of the cell surface of vertebrate neurons [12]. They are crucial for the proper function and maintenance of both the central and peripheral nervous system, particularly for the establishment of axon–myelin interactions [13]. Mice with a disrupted *B4galnt1* gene (*B4galnt1*-null) exhibit significant alterations in ganglioside profiles that may affect lipid raft composition and function. Instead, they synthesize the simpler gangliosides GM3 and GD3 and show progressive motor behavioral neuropathies, axon degeneration and demyelination without major histological defects at birth [14,15,16]. Interestingly, the behavioral differences observed in *B4galnt1*-null mice are not reflected in dendritic and spinal changes in the cortical pyramidal neurons [13,17].

In addition, cuprizone treatment, a method for inducing demyelination [18], can also affect the distribution and function of lipid rafts. Cuprizone (IUPAC name: N,N’-bis(cyclohexylideneamino)oxamide) is a copper chelator commonly used to induce demyelination in the central nervous system of mice. It serves as a valuable model for studying the pathophysiology of multiple sclerosis (MS) and other demyelinating diseases [19]. Administration of cuprizone leads to selective oligodendrocyte apoptosis in the CNS, resulting in degradation of the myelin sheath without invoking an adaptive immune response. This demyelinating effect is attributed to the compound’s ability to interfere with mitochondrial copper metabolism, which is crucial for cellular energy production and antioxidative defense mechanisms [20,21]. The resulting mitochondrial stress and dysfunction in oligodendrocytes accelerate myelin loss, mimicking aspects of demyelinating diseases.

Given these considerations, this study aims to understand the age-dependent effects of inhalational anesthetics on two different models of demyelination. We hypothesize that since *B4galnt1*-null mice, which lack complex gangliosides, and cuprizone-treated mice both experience demyelination, they will show a more pronounced age-dependent response to anesthetics. The disruption of myelin sheaths and the possible reorganization of lipid rafts in demyelinated regions could alter the membrane environment and affect the distribution and function of anesthetic targets, including ion channels and receptors localized in lipid rafts.

## 2. Materials and Methods

### 2.1. Animals

The present study was approved by the Ethics Committee of the Faculty of Medicine of the Josip Juraj Strossmayer University of Osijek (approval number: 2158-61-07-20-107). All experimental procedures complied with the ethical standards of the regional ethics committees and were conducted in accordance with institutional and regulatory guidelines to ensure humane treatment of the animals involved in the study.

A total of 16 C57BL/6 mice (WT) were used in this study, which were divided into two age groups: 12 mice were 6 months old and 4 were 12 months old. We also used 16 *B4galnt1*-null mice (KO), 3 mice at 6 months of age and 13 mice at 12 months of age. In addition, 14 C57BL/6 mice were fed cuprizone (WT + CPZ); this group included 6 mice at 6 months of age and 8 mice at 12 months of age. Cuprizone-fed mice (WT + CPZ) were fed a diet containing 0.2% cuprizone (Sigma-Aldrich, St. Louis, MO, USA) mixed into their powdered chow for 19 days. Before and after the diet, the animals were subjected to various motor and cognitive tests. During the experiment, all mice were kept in a 12 h light–dark cycle, with the light turned on at 7:00 a.m.

### 2.2. Basso–Beattie–Bresnahan (BBB) Locomotor Rating Scale

All animals in the study were assessed prior to induction of anesthesia using the modified Basso–Beattie–Bresnahan (BBB) locomotor rating scale [22]. The gross motor status of a mouse was scored from 0 to 2, with a maximum total score of 22, with each of the following features evaluated and scored individually: 1. plantar step; 2. trunk stability; 3. tail lifting; 4. predominant paw position; 5. movements of left hind paw–left forelimb; 6. resistance against the surface; 7. gait stability; 8. dorsal step; 9. toe spread; 10. position of the hind legs; 11. trunk tremor.

### 2.3. General Anesthesia

The volatile anesthetics isoflurane (Forane, Baxter Healthcare Corporation, Deerfield, IL, USA) and sevoflurane (Baxter, Deerfield, IL, USA) were used in the tests. For the administration of anesthesia, we used a system of linearly connected components that included an oxygen tank, a flow meter, a MATRX VIP 3000 vaporizer (Stoelting Co., Wood Dale, IL, USA), an introduction chamber, and a Fluovac Scavenger System (Stoelting Co., Wood Dale, IL, USA) as an absorber for the exhaust gasses. The oxygen flow was set to 400 mL. The concentration of anesthetic during the induction phase was 2 vol% for isoflurane or 3.4 vol% for sevoflurane. For the maintenance of anesthesia, the concentration was set to 1 vol% for isoflurane and 1.7 vol% for sevoflurane. The depth of anesthesia was determined on the basis of the withdrawal reflexes; in particular, the ear pinch and toe pinch reflexes. The animals continued to breathe spontaneously during anesthesia. The animals were anesthetized for 5 min. After the 5th min, the mice were exposed to room air for the next 4 min to ensure complete awakening. Anesthesia was performed at 24 h intervals between inductions.

### 2.4. Motor Tests

The tests were performed on the same day in a specific order: pre-test before induction of anesthesia; then induction of anesthesia; and after the animals were fully awake and their mobility had fully returned, the post-tests were performed.

Hindlimb extension reflex test. In the hindlimb extension reflex test, the animals are held by the tail in a vertical position for 10 s and the position of the hind legs is observed. The position of the hind legs was scored from 0 to 4 points: 0 points (one or both hindlimbs are paralyzed), 1 point (the hindlimbs are close to the body, the paws are closed and the reflexes are absent), 2 points (the leg is flexed but the reflexes are absent), 3 points (the animal spreads its legs at an angle of less than 90 degrees) and 4 points (the animal spreads its legs at an angle of more than 90 degrees).

Forelimb grip strength test. In the forelimb grip strength test, the mouse is placed with its front paws on a 3 mm thick wire at a height of 60 cm. The time during which the mouse holds on to the wire with both forelimbs is recorded. The maximum time that the mouse holds on to the wire is 30 s.

Rotarod test. The rotarod device (RotaRod, Ugo Basile, Gemonio, Italy) was used to test motor coordination and balance. It consists of five cylinders with a diameter of 3 cm, which are operated by a motor control that allows them to rotate at different speeds and accelerations. In this study, a fixed speed mode of 20 rpm was used up to the maximum allowed time of 3 min for training and 5 min for the experiment. The animals were trained on the rotating cylinder for 3 days before the test. If the animal fell off the rotating cylinder during the test, a latency period was measured as a measure of the maintenance of balance and coordination of movement.

### 2.5. Cognitive Tests

The tests were performed on the same day in a specific order: pre-test before induction of anesthesia; then induction of anesthesia; and after the animals were fully awake and their mobility had fully returned, the post-tests were performed.

Modified Lashley III maze. The modified Lashley III maze is a Plexiglas cage with a styrofoam labyrinth construction inside. The mice are offered food 24 h before the test. The mice have 20 min to become familiar with the maze. The mice in this test use their memory to search for the food. They have 900 s to find the hidden food along a specific path. The time needed to actively search for the food is the latency time and is a measure for this test.

Passive avoidance test. The passive avoidance task was performed in a training cage (Passive Avoidance, Ugo Basile, Gemonio, Italy) consisting of two sections, a light and a dark compartment, separated by a guillotine door. The doors close automatically when the mouse enters the dark compartment. Three seconds after entering the dark compartment, the mouse is stimulated with a short electric shock of 0.5 mA for 2 s through the wire mesh floor. The electrical stimulation can be felt on the mouse’s paws. The test checks fear and motivation. The latency time is the time taken for the mouse to enter the dark room and is an indicator of instinctive reactions.

### 2.6. Immunohistochemistry

For the immunohistochemical analysis, 12-month-old animals from all three groups—WT, WT + CPZ and KO—were used. Behavioral tests were previously carried out with these animals. To perform this analysis, the animals were deeply anesthetized, followed by cardiac perfusion with 0.1 M phosphate buffer saline (PBS) and 4% paraformaldehyde (Sigma-Aldrich, St. Louis, MO, USA) in 0.1 M PBS, pH 7.4. Dissected brains were fixed in 4% paraformaldehyde for 24 h and cryoprotected in a 10% and 20% sucrose solution in 0.1 M PBS. The samples were then frozen in 2-methylbutane (Sigma-Aldrich, St. Louis, MO, USA) at −80 °C and stored at the same temperature until further processing.

The frozen brain samples were sectioned on a cryostat (Leica, CM3050S, Nussloch, Germany) in the coronal direction with a thickness of 35 µm. Highly specific IgG monoclonal antibodies were used to detect the complex gangliosides (GM1, GD1a, GD1b and GT1b) (kindly provided by Ronald L. Schaar, MD, PhD, Department of Pharmacology, The Johns Hopkins School of Medicine (Baltimore, MD, USA)) [23]. In addition, monoclonal antibodies were used to detect myelin-associated glycoprotein (MAG) (Chemicon, Temecula, CA, USA), myelin basic protein (MBP) (QED Bioscience Inc., San Diego, CA, USA) and cyclic nucleotide phosphodiesterase (CNPase) (Abcam, Cambridge, CB2 0AX, UK). MAG, MBP and CNPase are markers for myelinated axons, and their use is aimed at determining the general degree of demyelination in the mice studied. Free-floating sections of 35 μm thickness were pretreated with a 1% hydrogen peroxide solution (Kemika, Zagreb, Croatia) in 0.1 M PBS to remove endogenous peroxidase activity. Nonspecific antibody binding was blocked using a solution of 1% bovine serum albumin (Sigma-Aldrich, St. Louis, MO, USA) and 5% goat serum (Gibco™ Goat Serum, New Zealand origin, Thermofisher Scientific, Waltham, MA, USA) in 0.1 M PBS for 2 h at 4 °C with continuous shaking. Incubation with the primary antibodies was performed overnight at 4 °C. All primary antibodies were prepared in the blocking solution and used at the following dilutions: anti-GM1, 1:3000; anti-GD1a, 1:10,000; anti-GD1b, 1:10,000; anti-GT1b, 1:3000; anti-MAG, 1:500; anti-MBP, 1:500; anti-CNPase, 1:500. After incubation, the sections were washed three times in 0.1 M PBS and incubated with the secondary antibody (biotinylated goat anti-mouse IgG; Jackson Immunoresearch lab, West Grove, PA, USA) diluted 1:500 in the blocking solution and incubated at 4 °C for 4 h. Sections were washed again and incubated in the A + B Vector Elite Kit (Vector Laboratories, Burlingame, CA, USA), prepared according to the manufacturer’s instructions, for 2 h at 4 °C. Sections were washed again and then developed using the Vector DAB Substrate Kit (Vector Laboratories, Burlingame, CA, USA), prepared according to the manufacturer’s instructions. Sections were coverslipped and imaged using the Zeiss Axiovert 200 M (Carl Zeiss, Jena, Germany). The digital images were quantified using ImageJ analysis software (version 1.52i, NIH).

### 2.7. Statistics

Statistical analysis was performed using Prism software version 10.2.3 (403) (GraphPad Software, Boston, MA, USA). The statistical significance level was set at *p* < 0.05. The normality of the data distribution was tested using the Shapiro–Wilk test. Since the data did not follow a normal distribution, non-parametric analyses were applied. To detect statistically significant differences between all groups, the Kruskal–Wallis test was applied, followed by Dunn’s multiple comparison test. The Mann–Whitney U test was used for pairwise comparisons between the isoflurane-treated and sevoflurane-treated groups. In the case of behavioral tests, hindlimb extension reflex tests, forelimb grip strength tests and passive avoidance tests, Kaplan–Meier survival analysis using the log–rank test (Mantel–Cox test) was used to determine statistically significant differences between groups.

## 3. Results

### 3.1. Pre-Test BBB Assessment Scale

**Figure 1:**
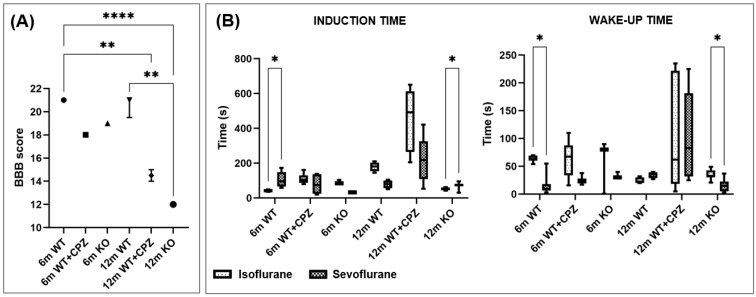
Assessment of the locomotor abilities of wild-type (WT), cuprizone-treated wild-type (WT + CPZ) and *B4galnt1*-null (KO) mice aged 6 and 12 months (6 m and 12 m). (**A**) Basso–Beattie–Bresnahan (BBB) assessment of the locomotor performance of the mice before motor and cognitive tests (median with interquartile range; Kruskal–Wallis test followed by Dunn’s multiple comparison test; ** *p* < 0.01, **** *p* < 0.0001); (**B**) Times necessary for the induction of and awakening from anesthesia with isoflurane or sevoflurane (minimum to maximum values; the line in the box represents the median; Mann–Whitney U-test; * *p* < 0.05).

The modified BBB locomotor test was used to assess the gross motor status of mice prior to the induction of anesthesia and behavioral testing. Data were analyzed using the Kruskal–Wallis test to determine differences between groups. The results (Figure 1A) show that KO mice aged 12 months had the lowest BBB score in all analyzed groups and WT mice aged 6 months had the highest score. Compared with the other groups, KO mice aged 12 months had a significantly lower pretest score than WT mice of the same age group (*p* < 0.01) and WT mice aged 6 months (*p* < 0.0001). WT mice aged 6 months had a significantly higher pretest BBB score than WT + CPZ mice aged 12 months (*p* < 0.01).

### 3.2. Induction of and Waking Up from Anesthesia

Anesthesia induction times. To determine whether there was a difference in the time required for the induction of anesthesia, we measured the time required to induce anesthesia in the animals with isoflurane and sevoflurane. The data were analyzed using the Kruskal–Wallis test to determine differences between the groups. Our results showed that WT + CPZ mice aged 12 months required the longest induction time with both anesthetics, although the difference in induction times between the two anesthetics was not significantly different. However, KO mice aged 12 months had a significantly longer induction time with sevoflurane compared with isoflurane (*p* < 0.05). The induction time was also longer with sevoflurane than with isoflurane in WT mice aged 6 months (*p* < 0.05). There was a trend toward shorter induction times with sevoflurane compared with isoflurane in WT + CPZ mice aged 6 months, KO mice aged 6 months and WT mice aged 12 months. The results are shown in Figure 1B.

Anesthesia wake-up times. A similar pattern was observed when the time required for the animals to wake up from anesthesia was measured (Figure 1B). WT + CPZ animals aged 12 months had the longest time to wake up from both anesthetics. A significant difference was observed in WT mice aged 6 months, whose wake-up time was significantly shorter after sevoflurane than after isoflurane (*p* < 0.05). The same was true for KO mice aged 12 months (*p* < 0.05), which also had shorter wake-up times after sevoflurane than after isoflurane. There was also a trend toward shorter wake-up times after sevoflurane in the other animal groups, except for WT mice aged 12 months.

### 3.3. Motor Tests

**Figure 2:**
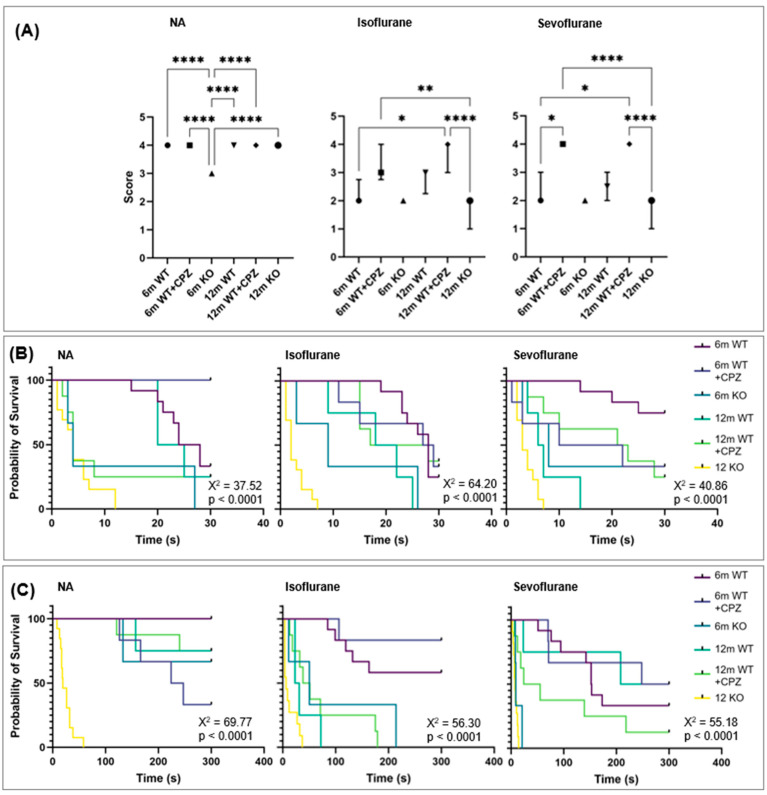
Analyses of behavioral tests of locomotor assessment in wild-type (WT), cuprizone-treated wild-type (WT + CPZ) and *B4galnt1*-null (KO) mice aged 6 and 12 months (6 m and 12 m). (**A**) Hindlimb extension reflex test in non-anesthetized mice (NA) and mice after anesthesia with isoflurane or sevoflurane (Kruskal–Wallis test followed by Dunn’s multiple comparison test; * *p* < 0.05, ** *p* < 0.01, **** *p* < 0.0001). Kaplan–Meier survival analysis using the log–rank test (Mantel–Cox test) to compare forelimb grip strength (**B**) and rotarod endurance (**C**) of non-anesthetized mice (NA) and mice after anesthesia with isoflurane or sevoflurane.

Hindlimb extension reflex test (HERT). To assess neuromuscular function in the mice, the HERT behavioral assessment was used (Figure 2A). Data were analyzed using the Kruskal–Wallis test to determine differences between groups. The results show that all groups of animals had similar scores prior to anesthesia, with the exception of KO mice aged 6 months. They had the lowest score and were significantly different from all other groups (*p* < 0.0001 in comparison with all groups). After induction with both anesthetics, WT + CPZ mice aged 12 months showed no change in their scores at 12 months of age. However, WT and KO mice of both age groups had lower values after both anesthetics. Notably, WT mice aged 6 months had a significantly lower score after isoflurane, which made them significantly different from WT + CPZ mice aged 12 months, whose scores did not change (*p* < 0.05). This group also showed significant differences after sevoflurane compared with WT + CPZ mice of both age groups (*p* < 0.05). In addition, WT + CPZ mice aged 6 months differed significantly from KO mice aged 12 months after both isoflurane (*p* < 0.01) and sevoflurane (*p* < 0.0001).

Forelimb grip strength test (FGST). The FGST was used to measure neuromuscular function and forelimb motor strength in the mice used in this study. Figure 2B shows a Kaplan–Meier survival analysis comparing the forelimb grip strength of mice before and after anesthesia with isoflurane or sevoflurane. Here, the mice that were able to hold on to the wire with both forelimbs for the entire duration of the test are considered censored, and those which were unable to do so are considered an event of interest. To determine differences between the groups, the data were further analyzed using the Kruskal–Wallis test (Figure S1A). The most striking observation is that KO mice aged 12 months showed the shortest survival time both before and after anesthesia, while WT mice aged 6 months showed the longest survival time. When comparing all groups before anesthesia, there was a significant difference between WT mice aged 6 months and KO mice aged 12 months in the loss of grip strength (*p* < 0.01). In addition, WT + CPZ mice aged 6 months had a significantly longer survival time than KO mice aged 12 months (*p* < 0.0001) and WT + CPZ mice aged 12 months (*p* < 0.05). After anesthesia, we observed an unexpected improvement in survival time in WT + CPZ mice aged 12 months, whereas survival time decreased in WT + CPZ mice aged 6 months and WT mice aged 12 months. After administration of both anesthetics (isoflurane and sevoflurane), the difference between WT mice aged 6 months and KO mice aged 12 months increased (*p* < 0.0001). The difference between WT + CPZ mice aged 6 months and KO mice aged 12 months decreased but remained significant (*p* < 0.0001). Since WT + CPZ mice aged 12 months showed improved survival times, their difference from KO mice aged 12 months became significant (*p* < 0.01).

Rotarod test. To assess the effects of the anesthetics on motor coordination and balance, we performed the rotarod test on all the groups of animals. Figure 2C shows a Kaplan–Meier survival analysis comparing the time animals spent on the rotarod device before and after anesthesia with isoflurane or sevoflurane. Here, mice that were able to remain on the rotarod for the entire duration of the test are considered censored, and those that were not able to remain are considered to have experienced an event of interest. Data were further analyzed using the Kruskal–Wallis test to determine differences between groups (Figure S1B). KO mice aged 12 months showed the lowest survival times on the rotarod, both before and after anesthesia, indicating significant deficits in motor coordination and balance due to the genetic modification. All other groups showed similar survival times before anesthesia. After isoflurane anesthesia, WT + CPZ mice aged 6 months showed a slight increase in survival time on the rotarod, with a significant difference compared with KO mice aged 12 months (*p* < 0.0001). Isoflurane had no effect on survival time on the rotarod in WT mice aged 6 months and KO mice aged 12 months, and the significant difference between them remained (*p* < 0.00001). Isoflurane affected the survival times of KO mice aged 6 months, WT mice aged 12 months and WT + CPZ mice aged 12 months. Sevoflurane had the most significant impact on KO mice aged 6 months, dramatically shortening their survival time on the rotarod. Survival was also shortened in WT + CPZ mice aged 12 months and WT mice aged 6 months after sevoflurane, but their significant difference compared with KO mice aged 12 months persisted (*p* < 0.00001). WT + CPZ mice aged 6 months showed a slight increase in survival time after sevoflurane, with a significant difference remaining compared with KO mice aged 12 months (*p* < 0.0001). The difference in rotarod survival time remained significant (*p* < 0.01) between KO and WT mice aged 12 months after anesthesia with isoflurane and sevoflurane, highlighting the severe motor impairments in KO mice.

### 3.4. Cognitive Tests

**Figure 3:**
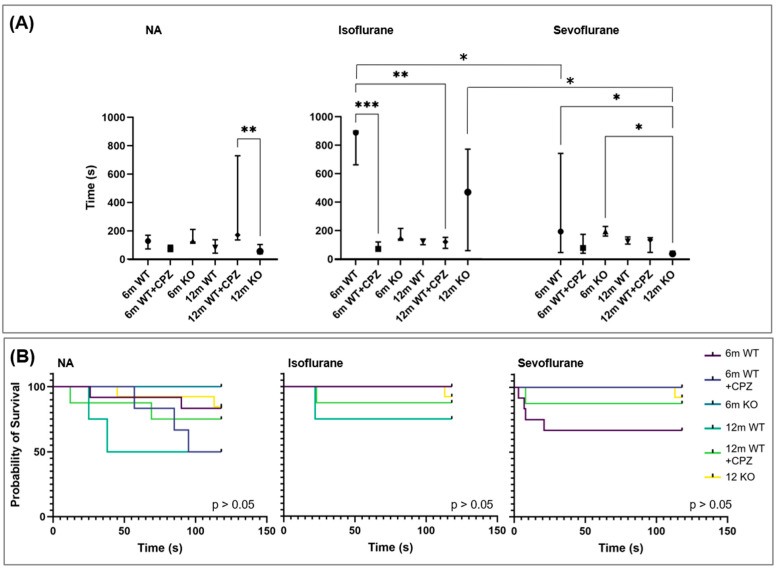
Analyses of cognitive behavioral tests in wild-type (WT), cuprizone-treated wild-type (WT + CPZ) and *B4galnt1*-null (KO) mice aged 6 and 12 months (6 m and 12 m). (**A**) Modified Lashley III maze test from non-anesthetized mice (NA) and mice after anesthesia with isoflurane or sevoflurane (Kruskal–Wallis test, followed by Dunn’s multiple comparison test for comparisons between groups with respect to the anesthetic used and the Mann–Whitney U test for pairwise comparisons between groups treated with different anesthetics; * *p* < 0.05, ** *p* < 0.01, *** *p* < 0.001). (**B**) The Kaplan–Meier survival analysis using the log–rank test (Mantel–Cox test) to compare the passive avoidance of non-anesthetized mice (NA) and mice after anesthesia with isoflurane or sevoflurane.

Modified Lashley III maze. To assess the effects of the anesthetics on spatial learning and memory, we used the modified Lashley III maze for all the animal groups. As explained earlier, the tests were performed on the same day—a pre-test immediately before induction of anesthesia and a post-test after the animals had fully recovered their motor functions. Figure 3A shows the analysis of the time required to complete the labyrinth before and after anesthesia with isoflurane or sevoflurane. The data were further analyzed using the Kruskal–Wallis test to determine differences between the groups. All animals tested had similar maze-solving times, with a significant difference observed between WT + CPZ mice aged 12 months and KO mice at the same age (*p* < 0.001).

After isoflurane anesthesia, WT mice aged 6 months showed the highest increase in maze-solving time and differed significantly from WT + CPZ mice aged 6 months (*p* < 0.0001) and WT + CPZ mice aged 12 months (*p* < 0.001). KO animals also spent more time solving the labyrinth after isoflurane anesthesia. Sevoflurane slightly increased the labyrinth-solving time in WT mice aged 6 months and WT + CPZ mice aged 12 months. Significant differences were found between WT mice aged 6 months and KO mice aged 12 months (*p* < 0.01) and between KO animals of both age groups (*p* < 0.01).

Passive avoidance test. To evaluate the effects of the anesthetics on learning and memory, we performed the passive avoidance test on all the animal groups. Figure 3B shows the Kaplan–Meier survival analysis of the latency to step-through in the passive avoidance apparatus before and after anesthesia with isoflurane or sevoflurane. Here, mice that were able to remain in the light compartment for the entire duration of the test were considered censored, and those that were unable to do so were considered to have experienced an event of interest. All the groups of animals had similar latency times prior to anesthesia, with the exception of the WT mice aged 12 months, which had slightly lower latency times. After administration of both anesthetics, the latency times increased in this group and were similar in all other animals tested. There was no significant difference in latency times between the test groups after isoflurane and sevoflurane anesthesia as determined by the Kruskal–Wallis test (Figure S2).

### 3.5. Immunohistochemistry

**Figure 4:**
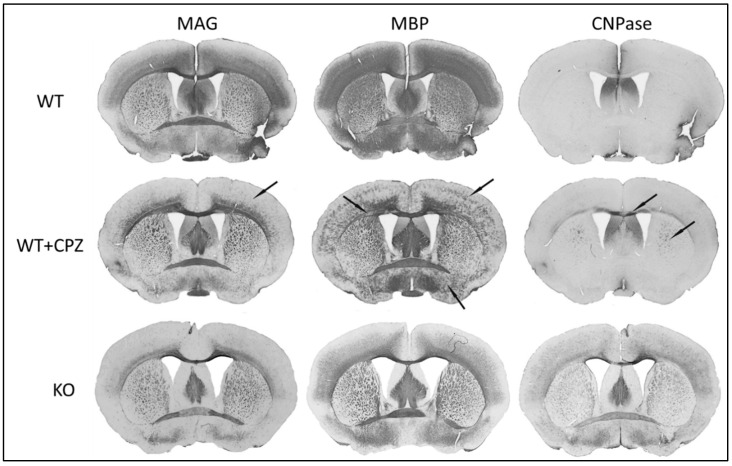
Immunohistochemical staining of myelination markers (MAG, MBP and CNPase) in the brains of wild-type (WT), cuprizone-treated wild-type (WT + CPZ) and *B4galnt1*-null (KO) mice on coronal sections of the forebrain. The arrows indicate the sites with the greatest differences in the distribution of the individual markers.

To assess the efficacy of cuprizone treatment in wild-type mice, we used myelin markers (anti-MAG, anti-MBP and anti-CNPase antibodies) to compare WT mice with WT + CPZ animals and with KO animals. Coronal sections of the forebrain at the level of the anterior commissure are shown in Figure 4. All three antibodies used—anti-MAG, anti-MBP and anti-CNPase—recognize epitopes on oligodendrocytes.

Figure 4 shows that KO mice have the lowest MAG levels. WT + CPZ mice also show a loss of MAG protein expression, particularly in the middle layers of the cortex and, to a lesser extent, in large fiber bundles such as the *corpus callosum* and anterior commissure. Dramatic changes can be observed in the expression of the MBP protein. The CNPase protein is only weakly expressed in the brains of WT mice, but its expression increases in WT + CPZ mice, especially in the large bundles of commissural fibers in the *corpus callosum*, the main migration pathway for adult oligodendrocyte progenitor cells. The phenomenon of increased CNPase expression is particularly pronounced in the brains of KO mice, where it is observed in all layers of the cortex and commissures.

**Figure 5:**
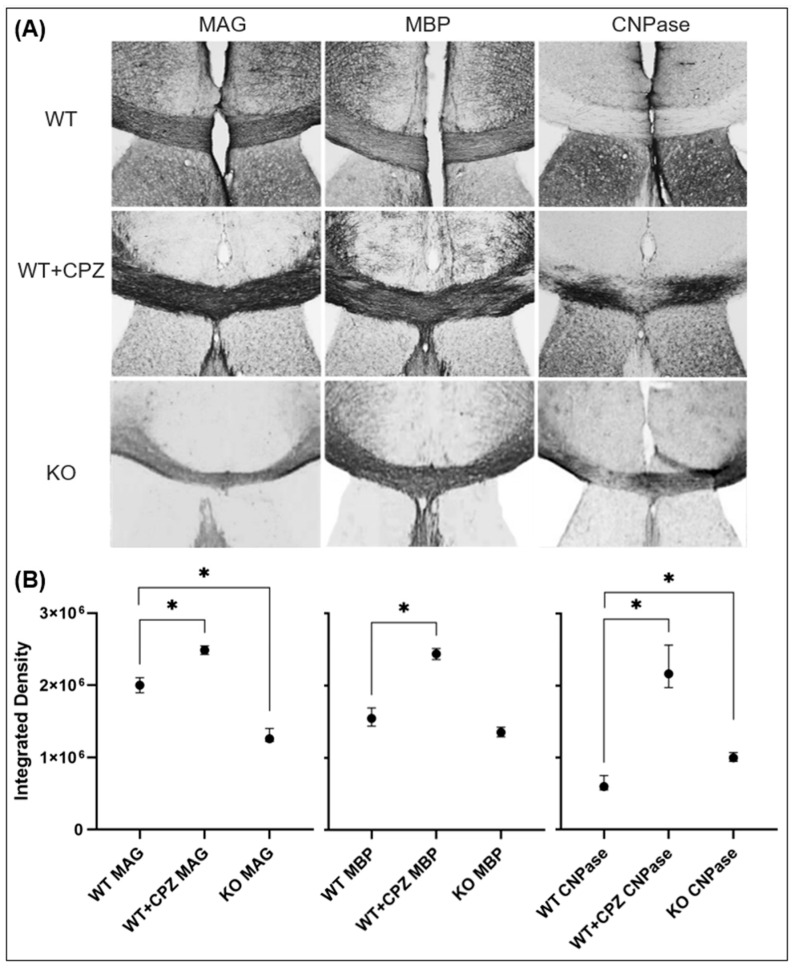
Expression level of myelination markers (MAG, MBP and CNPase) in the *corpus callosum* of the brains of wild-type (WT), cuprizone-treated wild-type (WT + CPZ) and *B4galnt1*-null (KO) mice. (**A**) Immunohistochemical staining of myelination markers (total magnification is 100×) and (**B**) their evaluation (median and interquartile range of integrated optical density). Mann–Whitney U test; * *p* < 0.05.

To further analyze the *corpus callosum*, we quantified the immunohistochemical staining of the myelin markers MAG, MBP and CNPase. As shown in Figure 5, our results indicate that MAG expression increases in WT + CPZ mice (*p* < 0.01) compared with WT mice, while it decreases in KO mice (*p* < 0.01). MBP expression changes from homogeneous staining in WT animals to a focal loss of staining in WT + CPZ animals, but its overall expression is increased (*p* < 0.01). MBP is not strongly altered in KO mice. CNPase showed the greatest change—its reactivity increased throughout the commissural bundle in both WT + CPZ (*p* < 0.01) and KO (*p* < 0.01) compared with WT mice, in which CNPase expression is very low.

**Figure 6:**
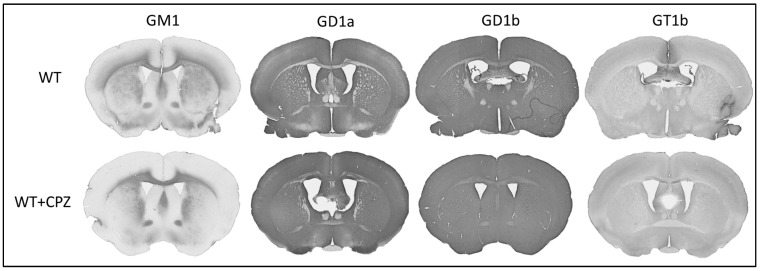
Immunohistochemical staining of the four major brain gangliosides (GM1, GD1a, GD1b and GT1b) in the brains of wild-type (WT) and cuprizone-treated wild-type (WT + CPZ) mice on coronal sections of the forebrain.

**Figure 7:**
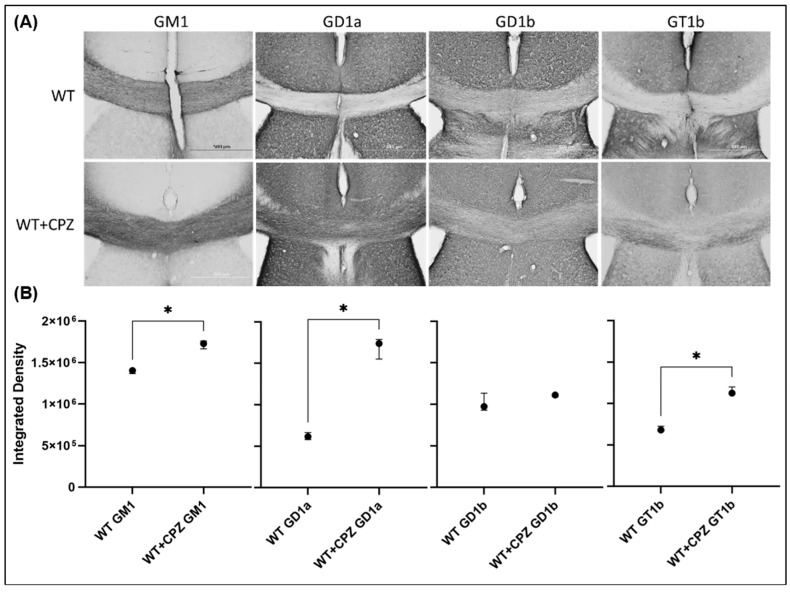
Expression level of the four major brain gangliosides (GM1, GD1a, GD1b and GT1b) in the *corpus callosum* of the brains of wild-type (WT) and cuprizone-treated wild-type (WT + CPZ) mice. (**A**) Immunohistochemical staining of gangliosides (total magnification is 100×) and (**B**) their evaluation (median and interquartile range of integrated optical density). Kruskal–Wallis test followed by Dunn’s multiple comparison test; * *p* < 0.05.

In addition to assessing the extent of demyelination by cuprizone treatment, we also investigated whether this treatment altered the distribution of gangliosides. Figure 6 shows the immunohistochemistry of coronal sections of forebrain from WT mice and WT + CPZ mice labeled with antibodies against the four most abundant brain gangliosides (GM1, GD1a, GD1b and GT1b), which are not present in KO mice. The overall distribution for all four antibodies is virtually identical in the forebrain (Figure 6). However, when we analyzed the *corpus callosum*, the quantification results showed a significant increase in three (GM1, GD1a and GT1b) of the four complex gangliosides (*p* < 0.01). Only ganglioside GD1b was not increased in WT + CPZ animals compared with WT animals (Figure 7).

## 4. Discussion

In this study, we investigated the sensitivity to inhalational anesthetics in two models of demyelination—congenital (*B4galnt1*-null mice) and chemically induced (cuprizone-treated mice). We were also interested in whether the response to anesthetics was age-dependent and therefore included mice aged 6 months (adult mice) and 12 months (old mice) in the study. Since demyelination and motor defects in *B4galnt1*-null mice manifest at 8 months of age, we did not include animals younger than 6 months in the study [15,16]. To assess the general motor status prior to anesthesia induction and behavioral tests, we subjected all animals to a modified BBB locomotor test [22]. As expected, older *B4galnt1*-null mice (aged 12 months) performed the worst, which is consistent with previous studies showing that these animals develop motor defects and demyelination with aging [15,16]. The lower BBB scores in these mice indicate severe neuromuscular impairment due to the lack of complex gangliosides, which are crucial for proper neuronal function and myelination [24,25]. Similarly, 12-month-old mice treated with cuprizone also showed reduced motor function, although their scores were slightly better than those of *B4galnt1*-null mice. Cuprizone, a copper chelator, induces demyelination by causing apoptosis of oligodendrocytes, resulting in myelin loss and subsequent motor deficits [18]. The better motor performance of cuprizone-treated mice suggests less severe demyelination or different compensatory mechanisms compared with *B4galnt1*-null mice.

Mice vary in their sensitivity to volatile anesthetics, which can be influenced by several factors such as genetic background, age, and the presence of underlying conditions such as demyelination. Our observations showed that 12-month-old mice treated with cuprizone required the longest induction time with both anesthetics. These mice also required the longest time to awaken from anesthesia, suggesting that the combined effects of aging and cuprizone-induced demyelination may reduce responsiveness to the anesthetics, requiring more time for effective induction and recovery. Interestingly, 12-month-old *B4galnt1*-null mice showed a significantly longer induction time with sevoflurane than with isoflurane. Since *B4galnt1*-null mice do not synthesize complex gangliosides, which are essential for maintaining membrane integrity and function, these molecules may play an important role in the mechanism of anesthesia. In addition, these *B4galnt1*-null mice woke up faster from sevoflurane; this may be due to its lower blood gas solubility coefficient, which allows for faster elimination from the body compared with isoflurane [26]. In adult WT mice, induction with sevoflurane also lasted longer than with isoflurane, suggesting that adult mice may metabolize or respond to sevoflurane differently than older or genetically modified mice. Considering the dynamics of ganglioside expression, which peaks in middle age and decreases with age, the effect of volatile anesthetics might follow this pattern and indirectly depend on it [27]. In addition, the amount of intracortical myelin, which is highest in middle age, could also influence induction times, as shown by MRI studies in humans [28,29]. In cuprizone-treated mice, 6-month-old *B4galnt1*-null mice and 12-month-old WT mice, induction times were shorter with isoflurane than with sevoflurane, suggesting that isoflurane may be more effective for faster induction due to its pharmacokinetic properties, which allow for a faster onset of action [26].

When we performed motor behavioral tests, our study showed that 12-month-old *B4galnt1*-null mice had the most severe motor deficits, as evidenced by the lowest scores on the hindlimb extension reflex, forelimb grip strength and rotarod tests—both before and after anesthesia with isoflurane or sevoflurane. These results are consistent with previous studies indicating that defects in ganglioside synthesis lead to demyelination and the subsequent deterioration of motor function [15,16]. The significant motor deficits observed in *B4galnt1*-null mice underscore the crucial role of gangliosides in maintaining neuronal function and myelin integrity [30]. WT mice treated with cuprizone at 12 months of age also showed motor deficits, but these were less severe than in the *B4galnt1*-null mice, probably due to the different mechanisms underlying demyelination in these models. The observation that hindlimb extension scores worsened after anesthesia with both isoflurane and sevoflurane in all groups tested is consistent with the known inhibitory effects of volatile anesthetics on reflex stimuli [31].

Six-month-old WT mice exhibited the longest duration in the grip strength test, indicating robust baseline neuromuscular function. As previously mentioned, this age group represents the peak of ganglioside expression and intracortical myelin levels, which are essential for optimal motor performance [12]. The administration of anesthetics resulted in different effects. The 12-month-old mice treated with cuprizone showed an increase in grip strength after anesthesia. Interestingly, 6-month-old mice treated with cuprizone showed a slight increase in rotarod performance after treatment with isoflurane, possibly indicating less pronounced depressive effects or an adaptive response specific to this group [32]. In general, anesthesia, especially with sevoflurane, did not significantly reduce the results of motor tests and, in some cases, even improved them, which is probably due to the effect of motor training.

In clinical practice, it has been noted that changes in memory and behavior often occur following exposure to volatile anesthetics, especially in the elderly. Recent studies suggest that long-term or repeated exposure to sevoflurane may promote apoptosis of hippocampal neurons and lead to cognitive changes [33,34]. In this study, all the animals tested had similar maze-solving times before anesthesia. However, after the administration of isoflurane, WT mice aged 6 months and *B4galnt1*-null mice aged 12 months showed a dramatic increase in the time required for maze solving, with the WT mice showing the most significant delay. These results suggest that volatile anesthetics, particularly isoflurane, significantly impair spatial learning and memory in younger WT mice and older *B4galnt1*-null mice. In WT mice aged 6 months and cuprizone-treated mice aged 12 months, sevoflurane slightly prolonged the labyrinth-solving times, suggesting a milder effect on cognitive function compared with isoflurane. In addition, we performed the passive avoidance test to examine the effects of anesthetics on learning and memory in all the groups of animals. All groups had similar latency times prior to anesthesia, with the exception of WT mice aged 12 months, which had slightly lower latency times. After the administration of both anesthetics, the latency times increased in this group and were consistent with those of the other animals tested. These results underscore the potential neurocognitive risks of volatile anesthetics, particularly with prolonged or repeated exposure.

To achieve comparable demyelination to that in *B4galnt1*-null mice, WT mice were treated with cuprizone, which effectively mirrors demyelination, including loss of the myelin proteins MBP and MAG. Demyelination does not affect all brain regions equally in the two mouse models studied, which could also explain the differences in the effects of the two anesthetics. Studies in rat models suggest that demyelination is more pronounced in the cerebral cortex than in the subcortical white matter, which we also confirmed with anti-MAG and anti-MBP antibodies. Interestingly, the large fiber bundles in the brainstem show no significant changes in myelin protein [18,21]. Our results showed that *B4galnt1*-null mice have the lowest MAG levels, which is consistent with previous findings [35]. Cuprizone-treated mice exhibited a marked loss of MAG expression, particularly in the middle layers of the cortex and, to a lesser extent, in large fiber bundles such as the *corpus callosum* and anterior commissure. Cuprizone induces demyelination by targeting oligodendrocytes, leading to their apoptosis and the subsequent loss of myelin proteins, including MAG. The pattern of MAG reduction suggests that cuprizone treatment disrupts the myelin sheath and particularly affects regions with high myelin turnover or regenerative requirements [18,19]. Quantification revealed an increase in MAG expression in cuprizone-treated mice compared with WT mice, which may indicate a compensatory response to the initial myelin damage that aims to stabilize and repair myelin sheaths. This adaptive response could be an attempt by the surviving oligodendrocytes to promote myelin regeneration and maintain axonal integrity. Another explanation is that the initial demyelination in these structures has led to increased access/availability of these proteins to antibodies. This hypothesis is supported by the presence of GD1a and GT1b in the same fibers. Functional studies have shown that these two gangliosides are receptors for MAG [36], which was questionable as there was no reactivity immunohistochemically to monoclonal antibodies directed against GD1a and GT1b in the large fiber bundles in wild-type mice. The presence of these gangliosides on fibers affected by demyelination may therefore be evidence that these epitopes exist and are accessible to highly specific monoclonal antibodies directed against them. We have also observed similar results with MBP. Our results show that the WT animals exhibited homogeneous MBP staining, while the cuprizone-treated animals showed focal MBP staining, indicating areas of myelin loss, but with an overall increase in MBP expression. This supports the assumption that demyelination only increased access for antibodies and did not decrease the expression of this epitope. The focal staining suggests localized demyelination caused by cuprizone treatment, which is known to induce apoptosis of oligodendrocytes and patchy demyelination [18]. Despite the localized losses, the overall increase in MBP expression could be attributed to compensatory upregulation in response to the initial myelin damage, representing an attempt by surviving oligodendrocytes to enhance remyelination and restore myelin integrity [19]. The unchanged MBP levels in *B4galnt1*-null mice underscore the chronic nature of their demyelination and reflect a different pathophysiology [16].

The 2′,3′-cyclic nucleotide 3′-phosphodiesterase (CNPase) enzyme is essential for the formation and maintenance of the myelin sheath. It marks the early differentiation of oligodendrocytes and plays a crucial role in cytoskeletal interactions and myelin integrity [37]. Low CNPase levels in WT mice indicate the normal maintenance of myelin without significant turnover or repair. Recent studies suggest that CNPase cooperates with and counteracts MBP in maintaining the cytoplasmic channels that support oligodendrocyte metabolism [38]. The expression of CNPase increased significantly in cuprizone-treated mice, particularly in the commissural fibers of the *corpus callosum*, indicating a robust response of oligodendrocytes to cuprizone-induced demyelination and active myelin repair and oligodendrocyte proliferation [39]. *B4galnt1*-null mice showed a pronounced increase in CNPase expression in all layers of the cortex and commissures, indicating extensive oligodendrocyte activity in response to chronic demyelination due to the absence of complex gangliosides [16,37].

The composition of lipid rafts somehow plays a role in these molecular differences. This is particularly pronounced in *B4galnt1*-null mice, as they do not synthesize complex gangliosides that are critical for lipid raft integrity and functionality. As integral components of lipid rafts that are essential for the formation of nodes of Ranvier, gangliosides play a critical role in the propagation of the action potential along the myelinated axons [40]. The gangliosides GM1 and GD1a, which are abundant in the nodes of Ranvier and paranodal regions, stabilize the node structure and cluster the ion channels [41]. Early studies using ganglioside-binding ligands showed the localization of GM1 at the nodal axolemma and at the membranes of Schwann cells [42]. Subsequent immunohistochemical studies confirmed the presence of gangliosides such as GM1, GD1a and GQ1b at the nodal gap and paranodal structures [40]. This localization is important because anti-ganglioside antibody-mediated injury to the node can lead to paralytic features of autoimmune neuropathies [43]. In *B4galnt1*-null mice, disruption of ganglioside synthesis likely alters the organization and function of nodes of Ranvier and impairs neuronal signaling [15,40]. The reduction or absence of specific gangliosides in these mice impairs the integrity of the nodes, resulting in reduced nerve conduction velocity and increased susceptibility to demyelination [41]. Further studies should investigate the mechanisms of changes in ganglioside distribution and their effects on lipid raft functionality in order to better understand the mechanisms of demyelination and improve treatment strategies. We also investigated whether treatment with cuprizone affects ganglioside distribution. Quantitative analysis of the *corpus callosum* revealed a significant increase in GM1, GD1a and GT1b expression in cuprizone-treated mice, suggesting that cuprizone alters ganglioside composition or access for antibodies. This probably affects the composition of lipid rafts. However, this should be confirmed at the level of lipid rafts, for example, by using super-resolution microscopy.

In conclusion, demyelination significantly affects anesthetic induction, emergence, and overall motor and cognitive performance. It also alters glycolipid epitopes, which are critical for understanding pathophysiology and thus warrant further research. Our study showed that isoflurane was more effective for induction and awakening, while sevoflurane resulted in fewer motor and cognitive side effects. However, our study had limitations, such as the small number of animals in certain groups and the inclusion of both sexes, which contributed to the variability in the behavioral data. Future research should investigate possible sex-specific responses and the role of myelin lipid rafts. This could lead to therapies that support remyelination and improve outcomes for patients with demyelinating diseases, thereby improving anesthetic management and treatment strategies.

## Data Availability

The raw data supporting the conclusions of this article will be made available by the authors on request.

## Supplementary Materials

The following supporting information can be downloaded at: https://www.mdpi.com/article/10.3390/cimb46080494/s1, Figure S1: Analyzes of the forelimb grip strength test (A) and rotarod endurance (B) in non-anesthetized mice (NA) and mice after anesthesia with isoflurane or sevoflurane (Kruskal-Wallis test followed by Dunn’s multiple comparison test, * *p* < 0.05, ** *p* < 0.01, *** *p* < 0.001, **** *p* < 0.0001). KO—*B4galnt1*-null mice, WT—wild-type mice, WT + CPZ—wild-type mice treated with cuprizone; Figure S2: Analysis of the passive avoidance test in non-anesthetized mice (NA) and mice after anesthesia with isoflurane or sevoflurane (Kruskal-Wallis test followed by Dunn’s multiple comparison test; there were no significant differences). KO—*B4galnt1*-null mice, WT—wild-type mice, WT + CPZ—wild-type mice treated with cuprizone.
