# Supplementary material for: Age-Related Effects of Inhalational Anesthetics in B4galnt1-Null and Cuprizone-Treated Mice: Clinically Relevant Insights into Demyelinating Diseases
Source: Curr Issues Mol Biol. 2024 Aug 1;46(8):8376–94. doi: 10.3390/cimb46080494 (PMC11352286; doi:10.3390/cimb46080494)
Supplement: Supplementary file 1 [file cimb-46-00494-s001.zip › cimb-3108735-supplementary.pdf]

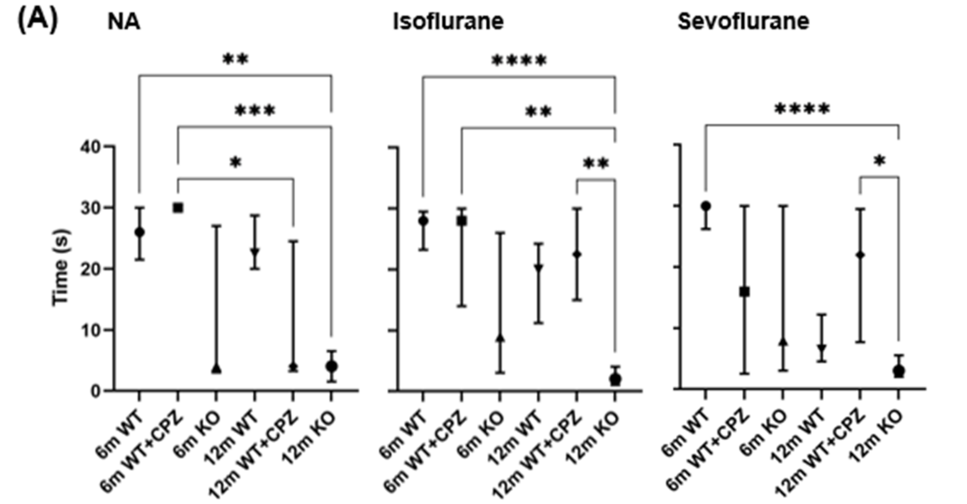

Kruskal-Wallis test	NA<br>p < 0.0001	Isoflurane<br>p < 0.0001	Sevoflurane<br>p < 0.0001
Post hoc Dunn's test	6m WT vs 12m KO<br>p = 0.002<br>6m WT+CPZ vs 12m WT+CPZ<br>p = 0.028<br>6m WT+CPZ vs 12m KO<br>p < 0.0001	6m WT vs 12m KO<br>p < 0.0001<br>6m WT+CPZ vs 12m KO<br>p = 0.001<br>12m WT+CPZ vs 12m KO<br>p < 0.001	6m WT vs 12m KO<br>p < 0.0001<br>12m WT+CPZ vs 12m KO<br>p = 0.035

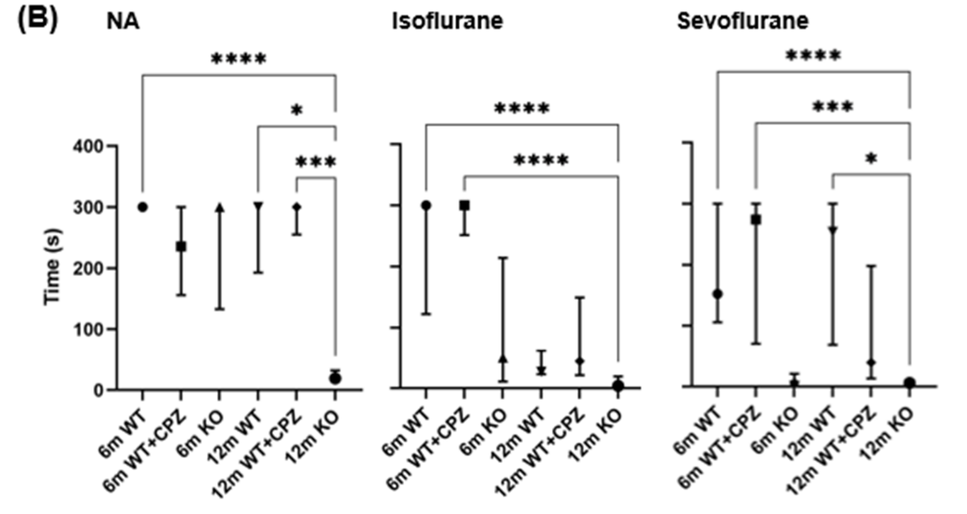

Kruskal-Wallis test	NA<br>p < 0.0001	Isoflurane<br>p < 0.0001	Sevoflurane<br>p < 0.0001
Post hoc Dunn's test	6m WT vs 12m KO<br>p < 0.0001<br>12m WT vs 12m KO<br>p = 0.019<br>12m WT+CPZ vs 12m KO<br>p < 0.001	6m WT vs 12m KO<br>p < 0.0001<br>6m WT+CPZ vs 12m KO<br>p < 0.001	6m WT vs 12m KO<br>p = 0.002<br>6m WT+CPZ vs 12m KO<br>p = 0.007

Figure S1. Analyzes of the forelimb grip strength test (A) and rotarod endurance (B) in non-anesthetized mice (NA) and mice after anesthesia with isoflurane or sevoflurane (Kruskal-Wallis test followed by Dunn’s multiple comparison test, * - p < 0.05, ** - p < 0.01, *** - p < 0.001, **** - p < 0.0001). KO - *B4galnt1*-null mice, WT - wild-type mice, WT + CPZ - wild-type mice treated with cuprizone.

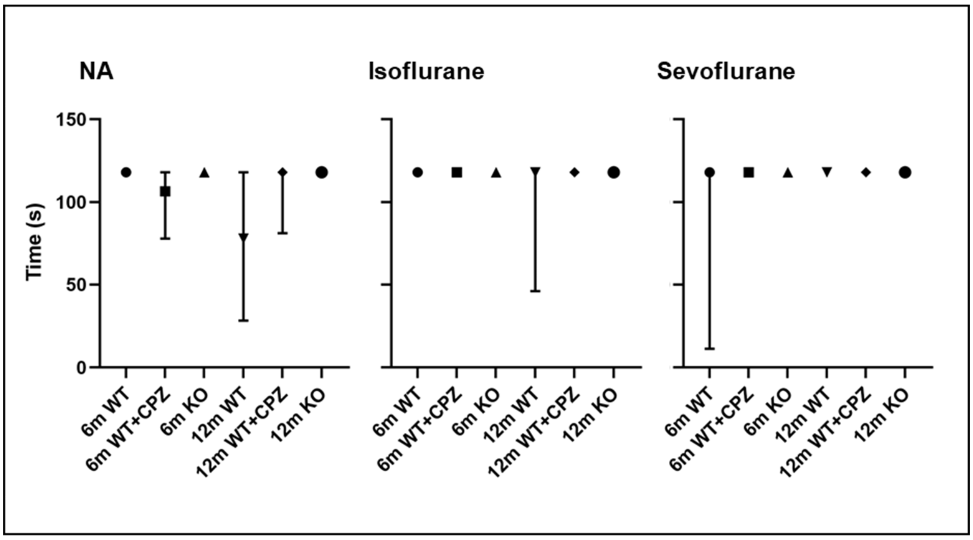

Figure S2. Analysis of the passive avoidance test in non-anesthetized mice (NA) and mice after anesthesia with isoflurane or sevoflurane (Kruskal-Wallis test followed by Dunn's multiple comparison test; there were no significant differences). KO - *B4galnt1*-null mice, WT - wild-type mice, WT + CPZ - wild-type mice treated with cuprizone.
